# Draft Genome Sequence of Bacillus licheniformis Strain UASWS1606, a Plant Biostimulant for Agriculture

**DOI:** 10.1128/MRA.00740-20

**Published:** 2020-09-10

**Authors:** Julien Crovadore, Bastien Cochard, Damien Grizard, Romain Chablais, Marine Baillarguet, Morgane Comby, François Lefort

**Affiliations:** a Plants and Pathogens Group, Research Institute, Land, Nature, and Environment, HEPIA, HES-SO University of Applied Sciences and Arts Western Switzerland, Jussy, Geneva, Switzerland; b Proxis Développement, Levallois-Perret, France; c LMH Microbiology Expert, Groupe Roullier, Saint-Malo, France; University of Arizona

## Abstract

Bacillus licheniformis is a well-known industrial bacterium. New strains show interesting properties of biostimulants and biological control agents for agriculture. Here, we report the draft genome sequence, obtained with an Illumina MiniSeq system, of strain UASWS1606 of the bacterium Bacillus licheniformis, which is being developed as an agricultural biostimulant.

## ANNOUNCEMENT

Bacillus licheniformis is a Gram-positive, endospore-forming, saprophytic motile bacterium that commonly occurs in plants, soils (1), or even birds’ feathers (2). Current taxonomy shows that it is closely related to Bacillus subtilis (3). It is a well-known bacterium used for its enzymes and antibiotics in a wide range of industries (4, 5), and some strains have already demonstrated interesting features for agronomic applications, such as imparting increased resistance to salt-alkaline stress (6), presenting endophytic behavior with biocontrol properties (7), and promoting plant growth and demonstrating fungus-antagonizing properties (8, 9). Bacillus licheniformis UASWS1606 was isolated from an agricultural clay loam soil in Presinge, Geneva, Switzerland (10), and initially identified as Bacillus licheniformis by 16S rRNA gene Sanger sequencing.

DNA was extracted with a modified cetyltrimethylammonium bromide (CTAB) protocol (11) from a culture grown exponentially overnight in Luria-Bertani broth at 25°C from a single colony. A sequencing library was built with the TruSeq Nano DNA library preparation kit (Illumina, USA). Whole-genome sequencing was performed using a MiniSeq high-output kit within one Illumina MiniSeq run with a 2 × 151-bp paired-end read length, which produced 6,409,906 reads, resulting in 220× genome coverage. The overall quality metrics of the reads were assessed with FastQC v0.11.5 (12). Genome assembly was performed with the SPAdes genome assembler v3.13.0 (13) with a setting of “paired-end assembly, careful mode,” yielding 33 contigs (≥200 bp), ordered with BioEdit v7.0.5 (14), and analyzed with QUAST v4.6.3 (15) with the setting of "QUAST: skip contigs shorter than 200 bp." The total genome length is 4,370,390 bp, with a GC content of 45.69% and an *N*_50_ value of 1,154,533 bp. Automated gene annotation carried out with the NCBI Prokaryotic Genome Annotation Pipeline (PGAP) v4.1 (16) identified 4,396 coding sequences and 83 RNA genes, while RAST v2.0 (17), using the classic RAST annotation scheme, detected 4,677 coding sequences and 81 RNA genes. PlasmidFinder v1.3 (18) and plasmidSPAdes, both using default settings, did not detect any plasmids. RAST v2.0 (17) did not find any complete transposons or phages integrated. NCBI BLAST (19) showed that the whole 16S rRNA gene (1,456 bp) shared 99.93% identity with 7 Bacillus paralicheniformis strains and then 99.86% identity with 13 Bacillus licheniformis and 2 Bacillus paralicheniformis strains. According to the NCBI SRA Taxonomy Analysis Tool (STAT), based on raw sequencing read analysis, Bacillus licheniformis UASWS1606 shares 56.5% of its genome with Bacillus licheniformis, 18.7% with Bacillus paralicheniformis, and 17.6% with Bacillus haynesii. The annotation confirmed the absence of toxins and superantigens, as well as virulence and disease genes, allowing this bacterium to be considered for agronomic and environmental uses. Regarding its agronomic application, four genes of the auxin biosynthesis pathway and many protein-coding genes involved in the biocontrol process, such as genes for transporters, plant cell lytic enzymes, siderophores, and other secondary metabolites, are present. Like Bacillus licheniformis strain CKA1 (20), the presence of 15 genes related to phosphorus metabolism, including the Pho operon, suggests a strong ability to solubilize phosphate. Phenotypic profiling confirmed auxin synthesis and phosphate solubilization. Annotation also revealed 22 genes linked to nitrogen metabolism, some of which may increase plant growth rates and biomass production.

### Data availability.

This whole-genome shotgun project was deposited in DDBJ/EMBL/GenBank under the accession number JAAOWI000000000. The version described in this paper is the first version, JAAOWI010000000. Raw sequencing data sets have been deposited in the NCBI Sequence Read Archive (SRA) database under the accession number SRX7906581.
